# A screen for nuclear transcripts identifies two linked noncoding RNAs associated with SC35 splicing domains

**DOI:** 10.1186/1471-2164-8-39

**Published:** 2007-02-01

**Authors:** John N Hutchinson, Alexander W Ensminger, Christine M Clemson, Christopher R Lynch, Jeanne B Lawrence, Andrew Chess

**Affiliations:** 1 Center for Human Genetic Research, Massachusetts General Hospital, Harvard Medical School, 185 Cambridge St., Boston, MA-02114, USA; 2 Department of Biology, Massachusetts Institute of Technology, 77 Massachusetts Avenue, Cambridge MA-02139, USA; 3 Department of Cell Biology, University of Massachusetts Medical Center, 55 Lake Avenue North, Worcester, MA 01655, USA

## Abstract

**Background:**

Noncoding RNA species play a diverse set of roles in the eukaryotic cell. While much recent attention has focused on smaller RNA species, larger noncoding transcripts are also thought to be highly abundant in mammalian cells. To search for large noncoding RNAs that might control gene expression or mRNA metabolism, we used Affymetrix expression arrays to identify polyadenylated RNA transcripts displaying nuclear enrichment.

**Results:**

This screen identified no more than three transcripts; *XIST*, and two unique noncoding nuclear enriched abundant transcripts (*NEAT*) RNAs strikingly located less than 70 kb apart on human chromosome 11: *NEAT1*, a noncoding RNA from the locus encoding for *TncRNA*, and *NEAT2 *(also known as *MALAT-1*). While the two *NEAT *transcripts share no significant homology with each other, each is conserved within the mammalian lineage, suggesting significant function for these noncoding RNAs. *NEAT2 *is extraordinarily well conserved for a noncoding RNA, more so than even *XIST*. Bioinformatic analyses of publicly available mouse transcriptome data support our findings from human cells as they confirm that the murine homologs of these noncoding RNAs are also nuclear enriched. RNA FISH analyses suggest that these noncoding RNAs function in mRNA metabolism as they demonstrate an intimate association of these RNA species with SC35 nuclear speckles in both human and mouse cells. These studies show that one of these transcripts, *NEAT1 *localizes to the periphery of such domains, whereas the neighboring transcript, *NEAT2*, is part of the long-sought polyadenylated component of nuclear speckles.

**Conclusion:**

Our genome-wide screens in two mammalian species reveal no more than three abundant large non-coding polyadenylated RNAs in the nucleus; the canonical large noncoding RNA *XIST *and *NEAT1 *and *NEAT2*. The function of these noncoding RNAs in mRNA metabolism is suggested by their high levels of conservation and their intimate association with SC35 splicing domains in multiple mammalian species.

## Background

While the RNA transcriptome was once considered to be a faithful intermediate between genome and proteome, a greater appreciation of its scope and function has developed over the past several years. In particular, much recent attention has focused on small (<100 nucleotide) RNA species which significantly impact gene expression in eukaryotes, either by modulating transcript levels or the packaging of specific chromatin (reviewed in [1]). However, much larger noncoding RNAs (ncRNAs) also play fundamental roles in eukaryotic metabolism and development [2].

Like smaller RNAs, large ncRNAs (> 1 kb) can also modulate chromatin states and gene expression. In *Drosophila melanogaster*, two ncRNAs, *roX1 *and *roX2*, target the Male Specific Lethal (MSL) complex to the male *X*-chromosome, a crucial step in *Drosophila *dosage compensation [3]. In mammalian dosage compensation, the large *Xist *transcript along with its antisense counterpart, *Tsix*, are crucial in modulating the epigenetic state of X-linked loci (reviewed in [4]). After transcription, *Xist *spreads across the entire length of one of the two X-chromosomes in female cells, resulting in the transcriptional silencing of most other loci located on the inactive X [4,5]. Another large ncRNA, *NRON*, was recently shown to be involved in nuclear trafficking, through its interaction with a variety of nuclear import proteins [6].

A number of RNAs also localize to specific nuclear subdomains, such as the ten to thirty SC35 splicing domains localized within each mammalian cell (reviewed in [7,8]). SC35 domains comprise large (0.5–3 micron) structures defined by immunofluorescence to the spliceosome assembly factor SC35 [9]; this excludes the Cajal Bodies and smaller entities defined by the snRNP "speckle" staining pattern, but otherwise, SC35 domains overlap nuclear speckles, splicing factor compartments (SFCs), and interchromatin granule clusters (IGCs). Much debate has centered on the function and composition of these domains, either as sites of pre-mRNA metabolism and export [10-12] or storage sites of splicing factors [8,13]. While each SC35 domain is enriched in a large number of splicing factors and other factors involved in mRNA metabolism, a large pool of polyadenylated (poly(A)) and possibly nuclear retained [14] RNA also localizes to each domain [13,15], the identity of which has implications for the function of SC35 domains.

The function(s) of noncoding RNAs which act as chromatin modulators and nuclear scaffolds, depends on their localization to the nucleus. Unlike RNAs coding for nuclear proteins, which exit the nucleus when used as templates for protein synthesis, noncoding nuclear RNAs may exist solely within the nucleus throughout their lifetime. We reasoned that RNA transcripts with nuclear roles like *XIST *or comprising the poly(A) component of SC35 domains could be identified using a simple microarray approach to compare nuclear and cytoplasmic RNA populations. With this approach, we identified two evolutionarily conserved large noncoding transcripts that, like *XIST*, are enriched in human nuclei. Both nuclear enriched abundant transcripts (NEATs) are located on chromosome 11 in humans, less than 70 kb apart. Analysis of publicly available mouse transcriptome data confirms that the mouse homologs of these noncoding RNAs, located less than 50 kb apart on mouse chromosome 19, are also nuclear enriched. Each of these noncoding RNAs shows a broad distribution in different tissues, though their expression patterns differ slightly. Through RNA fluorescence *in situ *hybridization (FISH), we show that the mouse and human homologs of *NEAT1 *localize to the periphery of SC35 domains. Strikingly, the second transcript, *NEAT2 *localizes to the interior of all mature SC35 domains, suggesting a role for the transcript in either the structure or function of these nuclear substructures. Such pan-localization to SC35 domains by a noncoding poly(A) transcript has been discussed for several years [11,13,16,17], thus the identification of *NEAT2 *represents an important step towards the further molecular and functional dissection of these domains.

## Results

### An array-based approach to identify ubiquitously expressed nuclear RNAs

The classical long ncRNA with a nuclear role is *XIST*. *XIST *is a large, spliced, ncRNA, which is polyadenylated and stably expressed in female somatic cells where it localizes to the nucleus and tightly associates with one of the two X chromosomes [4]. Using *XIST'*s properties as a guide, we looked for noncoding RNAs with nuclear functions by searching for similarly nuclear-enriched noncoding transcripts.

**Figure 1:**
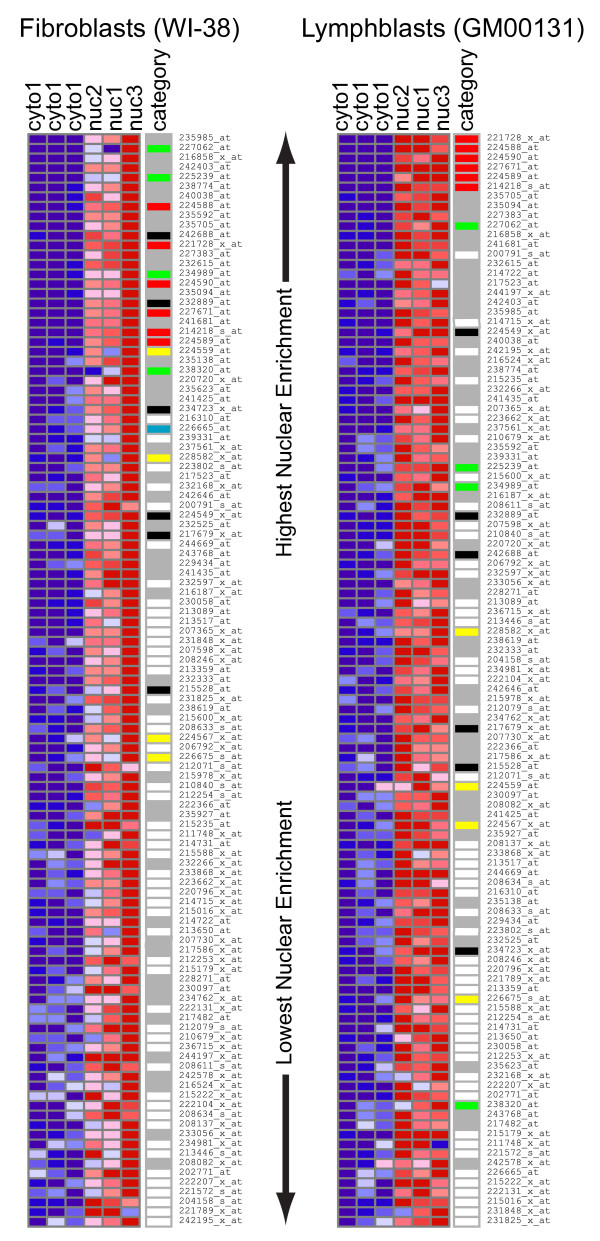
**Nuclear Enriched Probe Sets in Lymphoblasts and Fibroblasts**. Representative microarray hybridization of the 113 probe sets expressed above threshold that were more than 2 fold enriched in nuclear fraction samples (nuc1, nuc2 and nuc3) versus cytoplasmic fraction samples (cyto1, cyto2 and cyto3) on average in both fibroblasts (WI-38) and EBV-transformed lymphoblasts (GM00131). Probe sets are ranked in descending order of nuclear fraction enrichment from top to bottom. Probe expression is row normalized to show high expression as red and low expression as blue. Nuclear enriched probes were qualified into separate transcript type categories using the University of California at Santa Cruz genome browser and represented graphically beside the representative microarray hybridization. Probes to introns are represented in grey, protein coding transcripts (ORF containing) in white, the *XIST *locus in red, the *NEAT1 *locus in green and the *NEAT2 *locus in yellow.

To identify nuclear enriched transcripts, we utilized the Affymetrix U133A and U133B expression array set to compare the nuclear and cytoplasmic RNA fractions of two female cell types: the primary human fibroblast cell line, WI-38, and an Epstein-Barr Virus (EBV)-transformed lymphoblastoid line, GM00131. Based on the Unigene database (Build 133, April 20, 2001), these arrays allow the analysis of 39,000 transcripts with over 45,000 probes. In each cell line, many probe sets were enriched greater than 2-fold in the nuclear fraction, however only 113 of these were enriched in both (Figure 1 & see Additional file 1). Nuclear enriched probes were aligned to the human genome and their corresponding loci described using the University of California at Santa Cruz genome browser [19]. Based on these analyses, these abundant nuclear sequences included six probes to *XIST*, forty-two to introns, fifty-one to open reading frame (ORF) genes, six to repeat elements, and four probes each to two autosomal seemingly noncoding RNA loci.

The presence of a number of intronic probes on the Affymetrix arrays was striking, suggesting that a large number of Unigene EST clusters are not unique transcripts but instead represent improperly annotated introns. Indeed, many of these intronic probes had corresponding poly(A) repeats located in downstream genomic sequence which likely facilitated the annealing of oligo-dT primers at these sites during reverse transcription reactions. Affymetrix array design, which uses sequences selected from GenBank, dbEST and RefSeq, may not have completely filtered out such such falsely primed intronic-derived ESTs. Genes do sometimes reside within the introns of other transcripts [23,24], however, of the over 20 intronic probes we examined, all were transcribed from the same strand as the gene within which they reside. As there is no reported bias for intra-intronic genes to be transcribed in the same direction as the overlapping transcript, these data suggest that the vast majority of these probes detect intronic RNA rather than novel genes.

Nuclear enriched, non-intronic sequences aligned to either repeat elements and ORF or apparently non-ORF (noncoding) RNA transcripts. The enrichment of ORF genes within the nucleus may hold clues to RNA metabolism and may indicate high-level constitutive transcription coupled to relatively rapid cytoplasmic mRNA turnover. Perhaps the regulation of these genes is post-transcriptional, with other factors (such as microRNAs) regulating cytoplasmic RNA stability. Alternatively, they may be regulated by nuclear retention mechanisms similar to those allowing rapid translation of the mouse cationic amino acid transporter in response to stress [25].

To address these possibilities we used the GOStat program [26] to determine if any gene ontology subgroup was overrepresented in our sample of nuclear enriched genes. The 93 probes to coding sequences (excluding repetitive sequences and probes to noncoding RNAs) represent 87 unique genes of which 61 possess gene ontology annotations. Of these, 21 are nuclear components as compared to 5509 of 31046 in the genome as a whole, revealing a statistically significant enrichment of nuclear genes in this sample set (P = 0.0309). The biological significance of this finding is unclear but may reflect translation of these proteins either within the nucleus or within the perinuclear space [27], which may have copurified with the nuclear fraction.

Of the 3 noncoding transcripts identified, *XIST *is the most consistently nuclear-enriched ncRNA identified by this screen, as the six probes to it are enriched 7.8 to 11.3-fold in the nuclear fraction. Probes to two autosomal apparently noncoding RNA loci are also comparably nuclear enriched in both cell lines. We named these two loci *NEAT1 *and *NEAT2*, to reflect their status as nuclear enriched, abundant transcripts and their clustering within the genome (see below). Four probes to the first locus, *NEAT1*, are nuclear enriched from 2.1 to 25.6-fold in fibroblasts and lymphoblasts and four different probes to the *NEAT2 *locus show nuclear enrichment from 2.2 to 7.4-fold in fibroblasts and lymphoblasts. *NEAT1 *and *NEAT2 *were also as abundant as *XIST *as their probes showed a similar range of intensities as *XIST *probes.

### Two noncoding RNAs enriched in the nuclei of human cells

**Figure 2:**
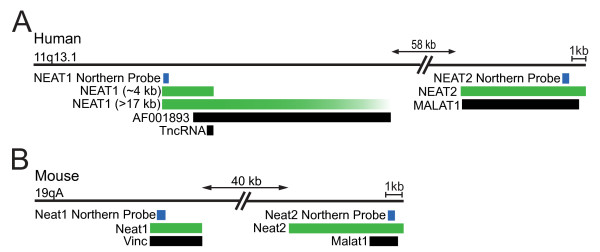
**Genomic Organization of the Human and Mouse *NEAT1 *and *NEAT2 *Loci**. (A, B) Graphical representation of locations and lengths of transcripts and probes mapping to the genomic region of (A) human *NEAT1*/*NEAT2 *on 11q13.1 and (B) mouse *Neat1*/*Neat2 *on 19qA. All transcripts run 5' to 3' from left to right. Transcripts represented in green (*NEAT1*, *NEAT2*, *Neat1 *and *Neat2*) were characterized by Northern analysis, 5'RLM-RACE and 3' RACE. Genbank accession number for these transcripts are: NEAT1 – EF177379, NEAT2 – EF177381, Neat1 – EF177378 and Neat2 – EF177380. For these transcripts, all 3' poly(A) ends had a consensus AAUAAA 10–30 nucleotides 5' of the poly(A) site and a GU rich and/or U rich element 3' of the site. Human *NEAT1 *is observed to extend past its primary poly(A) site, resulting in a larger, >17 kb transcript. The 3' end of this larger transcript is drawn fading to represent that while its location is suggested by AF001893 (Guru et al., 1997) and our own Northern analyses (data not shown), we were unable to fully map its 3' end by RACE, most likely due to a series of genomic poly(A) repeats throughout the transcript. Transcripts in black were previously described to various levels by EST walking, Northern and RACE analysis. Probes used in our Northern analyses (Figure 3) are represented in blue.

Genomic alignment, along with Northern blot and Rapid Amplification of cDNA Ends (RACE) analyses, defines the two NEAT transcripts as unique loci, separated by less than 60 kb on chromosome 11q13.1 of humans with no apparent intervening genes (Figure 2A). The genomic proximity of these two transcripts is striking, given that the human genome is roughly 3000 Mb and the probability of an unbiased screen returning two genes located side by side is less than 1 in 30,000. Transcription within the *NEAT1 *and *NEAT2 *loci has previously been described but not fully characterized [28-31]. Notably, there is no significant homology between the *NEAT1 *and *NEAT2 *loci (see below).

### *NEAT1 *is a large, infrequently spliced RNA

**Figure 3:**
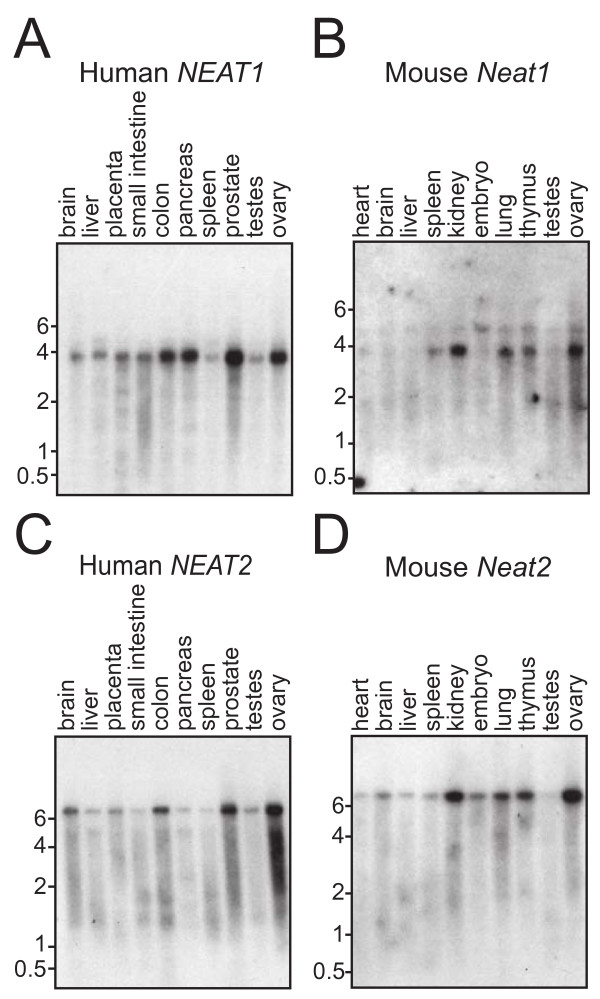
**Northern Analysis of *NEAT1 *and *NEAT2/MALAT-1 *in mouse and human tissues**. Radiolabeled probes to *NEAT1 *and *NEAT2/MALAT-1 *show a ~3.5 kb transcript for the human and mouse *NEAT1 *loci (A,C) and a transcript of more than 6 kb for the human and mouse *NEAT2 *loci (B,D). The analyses were carried out using First Choice Northern Blots (Ambion), which contain 2 micrograms each of poly(A) RNA from various tissues. Equal loading of RNA is ensured by Ambion for each lot of blots with either GAPDH or beta-actin probes for human or mouse blots respectively. Difficulties in transferring large transcripts to Northern blots may explain the absence of a larger (>17 kb), *NEAT1 *transcript (data not shown and [29]).

Our detailed molecular analysis by 5'-RLM/3'-RACE and cDNA sequencing indicates that the primary transcript from the NEAT1 locus is a predominantly unspliced, 3.7 kb, poly(A) ncRNA (Figure 2A & see Additional file 2) (sequence deposited in Genbank as EF177379). Northern blot analysis suggests a transcript ~4 kb in length with broad expression and highest levels in the ovary, prostate, colon and pancreas (Figure 3A). While this blot provided clear evidence of a widely expressed 4 kb transcript, transcripts above 9 kb in length may not have transferred efficiently to the membrane. A larger poly(A) transcript (>17 kb) (AF001892 and AF001893) with an overlapping 5' end is also suggested by other, less precise Northern data (data not shown and [29]). The size of this transcript is difficult to define, due likely to a combination of transcript heterogeneity and the difficulty of detecting large RNAs on Northern blots. Quantitative RT-PCR analysis suggests that this large transcript is expressed at significantly lower levels than the ~4 kb transcript (data not shown). While a small (<500 bases) cDNA clone, trophoblast-derived noncoding RNA (*TncRNA*), mapping to the 3' end of *NEAT1 *(~4 kb transcript) has also been described, it is exclusively expressed in trophoblasts [28,31]. Our results indicate the existence of at least two unique isoforms of *NEAT1*: a widely expressed, abundant 4 kb transcript and a much larger (> 17 kb), less common transcript sharing a transcriptional start site, based on 5'-RLM-RACE and Northern analysis.

### *NEAT2 *is a large, infrequently spliced RNA

Previously, a transcript in the *NEAT2 *region was partially described in a screen for genes overexpressed in metastatic non-small-cell lung cancer and given the name Metastasis Associated in Lung Adenocarcinoma Transcript 1 (*MALAT-1*) [30]. While *MALAT-1 *was initially defined as a cluster of ESTs more than 8 kb in length, our more precise 5'-RLM/3'-RACE studies of the *NEAT2 *locus show a slightly different sequence for the transcript which more closely matches the current database of ESTs (Figure 2A) (sequence deposited in Genbank as EF177381). As the functional relevance of this transcript to metastatic potential awaits further analysis, we will refer to this transcript as *NEAT2/MALAT-1*.

The vast majority of *NEAT2/MALAT-1 *is present in the cell in an unspliced form (see Additional file 2). Like *NEAT1*, *NEAT2/MALAT-1 *shows broad tissue expression, with highest expression in the ovaries, prostate and colon (Figure 3C), but unlike *NEAT1*, not the pancreas. Importantly, neither transcript (spliced or unspliced) has an open reading frame (ORF) of significant size. Despite the presence of antisense transcripts in the genomic annotation of the NEAT cluster, we detect no antisense transcription at either NEAT locus in GM00131 cells, HeLa cells or in the undifferentiated female human embryonic stem cell line H9 (NIH code WA09) by quantitative RT-PCR and Northern analysis (data not shown).

### Comparative genomic analysis of *NEAT1 *and *NEAT2*

The presence of two highly conserved areas within *NEAT1*, along with genomic synteny, facilitated the discovery of the mouse homolog of *NEAT1 *on chromosome 19 (Figure 2B). Detailed molecular analysis suggests that mouse *Neat1 *is a ~3.2 kb transcript with no significant ORFs (sequence deposited in Genbank as EF177378). Unlike human *NEAT1*, quantitative RT-PCR analysis does not detect any larger transcripts from the locus (data not shown). Moreover, the only sequence conservation between mouse and human is within this 3.2 kb transcript.

The mouse *Neat2 *locus is located approximately 40 kb from mouse *Neat1 *(Figure 2B). As with human *NEAT1*, murine *Neat1 *has no significant ORFs. While mouse *Malat-1 *was originally described as a series of overlapping ESTs homologous to human *MALAT-1 *[30], we have delineated the transcript from the mouse *Neat2 *locus by Northern analysis and 5'-RLM/3'-RACE (sequence deposited in Genbank as EF177380). Taken together, these data indicate that the mouse *Neat2/Malat-1 *transcript is ~7 kb, with broad tissue expression. Like human *NEAT2/MALAT-1*, mouse *Neat2/Malat-1 *has no significant ORFs.

To the extent that tissues overlap between blots, mouse *Neat1 *and *Neat2/Malat-1 *show similarly broad expression to human *NEAT1 *and *NEAT2/MALAT-1*, with highest levels in ovary, kidney, lung and thymus (Figure 3B and 3D). Significantly, while the sequence of both transcripts is conserved within the mammalian lineage, this conservation does not extend to any ORFs.

**Figure 4:**
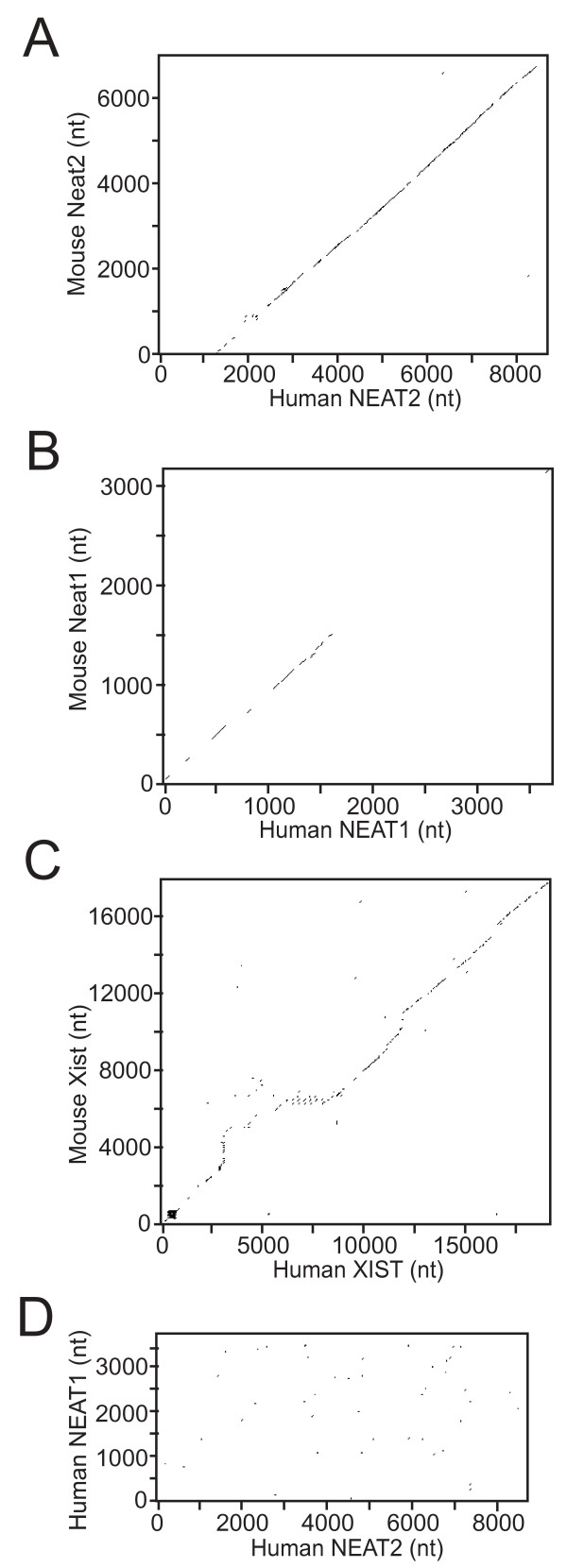
**Comparison of *NEAT1 *and *NEAT2/MALAT-1 *between the human and mouse genomes**. (A) The dot plot details the unspliced 8708 nt of the human *NEAT2/MALAT-1 *transcript as compared to the 6982 nt mouse *Neat2/Malat-1 *transcript and shows strong conservation across the length of the *NEAT2/MALAT-1 *transcript. (B) The dot plot details the 3729 nt human *NEAT1 *transcript as compared to the 3177 nt mouse *Neat1 *transcript. Two islands of relatively high identity were identified by this comparison: nucleotide position 483–597 of human *NEAT1 *and 450–565 of mouse *Neat1 *show 87% identity, and nucleotide position 1113–1368 of human *NEAT1 *and 1013–1238 of mouse *Neat1 *show 80% identity. (C) A dot plot with identical parameters between the primary human and mouse *XIST *transcripts is shown for comparison. (D) A dot plot between human *NEAT1 *and *NEAT2/MALAT-1 *with a 50% identity cutoff demonstrates the lack of conservation between the transcripts.

*NEAT2/MALAT-1 *is exceptionally conserved, considering that noncoding RNAs typically have less evolutionary constraints placed on their primary sequence than protein-coding genes [32] (Figure 4A). In particular, where insertions and deletions often disrupt ORFs by frame-shift mutations, noncoding RNAs are likely more tolerant of such changes, provided that they do not interfere with secondary structure or function. *NEAT2/MALAT-1 *does not contain the series of expanded repeats that are seen in *XIST *([33] and Figure 4C), suggesting that perhaps *NEAT2NEAT2/MALAT-1 *is less tolerant of such changes than *XIST*. Comparative genomic analysis indicates that *NEAT2/MALAT-1 *is conserved within multiple mammalian species, yet no *NEAT2/MALAT-1 *homologs were identified in any non-mammalian species. The presence of a *NEAT2/MALAT-1 *homolog in the non-eutherian opossum, *Monodelphis domestica*, together with the apparent absence of the transcript in non-mammalian species suggests that *NEAT2/MALAT-1 *is specific to the mammalian lineage. The strikingly high level of conservation of *NEAT2/MALAT-1 *within mammals suggests multiple constraints on its sequence. Like the conservation of other noncoding elements within the genome [34], this conservation awaits explanation through further analysis.

*NEAT1 *does not share the broad conservation seen for *NEAT2/MALAT-1 *between human and mouse (Figure 4B). Two small segments of the transcript are, however, conserved between humans and mice. Strikingly, the region most conserved between mouse and human *NEAT1 *is also the most conserved region between human *NEAT1 *and *NEAT1 *of dogs, rats, and cows (data not shown). Interestingly, *NEAT1 *is present on two different dog chromosomes, only one of which is clustered with the sole dog *NEAT2/MALAT-1*. Lack of synteny surrounding the *NEAT1 *paralogs suggests that a large-scale duplication within the dog lineage does not explain this event. Like *NEAT2/MALAT-1*, no non-mammalian homologs of *NEAT1 *were identified. Additionally, we cannot rule out the possibility that *NEAT1 *may be eutherian specific, due to gaps in the opossum genome around the putative NEAT cluster. As previously noted, dot plot analyses reveal that despite the genomic proximity of the *NEAT1 *and *NEAT2 *loci, their transcripts show no significant homology to each other (Figure 4D).

The identification of mouse homologs of *NEAT1 *and *NEAT2/MALAT-1 *allowed us to examine nuclear enrichment data from the Mouse Transcriptome Project [35]. This dataset was generated using Massive Parallel Signature Sequencing (MPSS), a technique which involves the sequencing based quantification of over 2 million short ESTs per sample [36]. EST populations of nuclear and post-nuclear subcellular fractionations were examined in the male mouse cell line BLK CL.4 and male mouse liver tissue. In both the BLK CL.4 cell line and mouse liver, both *Neat1 *and *Neat2/Malat-1 *were both more than ten-fold enriched in nuclear fractions (Table 1). Only four other transcripts (all of which are ORF genes) show nuclear enrichment levels similar to *Neat1 *and *Neat2/Malat-1*. These protein coding transcripts may be, like those seen in our microarray screen, regulated by various post-transcriptional mechanisms which result in their nuclear retention. Two-hundred and fifteen unique mouse genes were more than 2 fold enriched in both BLK CL.4 and liver nuclei (see Additional file 3) and of these 174 had gene ontology annotations. Similar to our human studies, of these annotated genes, 60 were nuclear components as compared to 5302 of 17217 genes in the murine genome as a whole, revealing a statistically significant enrichment of nuclear genes in this sample set (P = 0.00056). As noted above, this may reflect nuclear or perinuclear translation of these transcripts and copurification of the perinuclear component with the nuclear fraction. Notably, nuclear enrichment of both *Neat1 *and *Neat2/Malat-1 *is observed in mice using a distinct technology for expression analysis of different tissues than were used in our human studies.

Like *XIST*, *NEAT1 *and *NEAT2/MALAT-1 *appear to represent conserved nuclear-enriched, ncRNAs present within a diverse range of human and mouse cells. Taken together, these results suggest an important role for *NEAT1 *and *NEAT2/MALAT-1 *within the nuclei of mammalian cells.

### Subcellular localization of *NEAT1 *and *NEAT2/MALAT-1*

RNA FISH analyses confirmed the nuclear enrichment of both *NEAT1 and NEAT2/MALAT-1 *in a broad range of human and mouse cell lines. While we do not rule out the possibility of very low cytoplasmic levels, significant signal is not observed outside the nucleus; even using conditions optimized for cytoplasmic mRNA (see Methods). Both these transcripts are abundant and easily detectable. In interphase nuclei, both *NEAT1 *and *NEAT2/MALAT-1 *seem to avoid nucleoli and DAPI dense heterochromatin.

**Figure 5:**
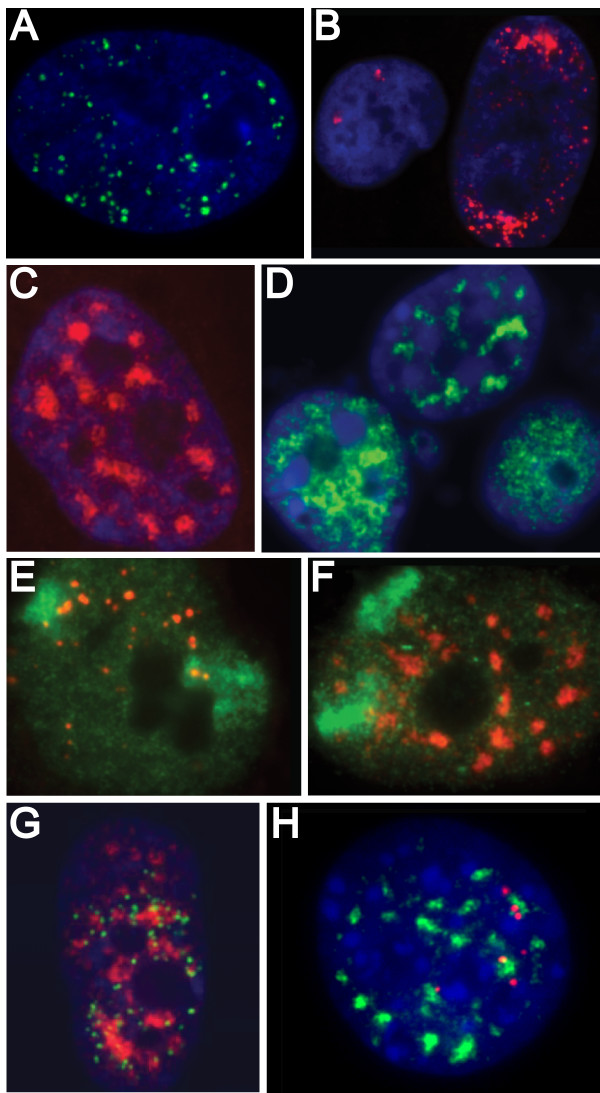
**Subcellular localization of human and mouse *NEAT1 *and *NEAT2/MALAT-1***. (A) Tig1 cells: Human *NEAT1 *RNA (green) distributes in discrete foci through the nucleus and is not detected in the cytoplasm. (B) Tig1 cells: *NEAT1 *RNA (red) is transcribed from 2 loci early in G1 (left) and occasionally shows a highly polarized pattern (right). (C) Tig1 cells: *NEAT2/MALAT-1 *RNA (red) is not detected in the cytoplasm at interphase and concentrates in domains in the nucleus with weaker signal apparent throughout the nucleoplasm. (D) NIH-3T3s: Mouse *Neat2/Malat-1 *RNA (green) localizes as bright nucleoplasmic signal in some cells and concentrates in domains in others. (E) *NEAT1 *RNA (red) does not localize to its parent chromosome 11 (green). (F) *NEAT2/MALAT-1 *RNA (red) rapidly disperses from its site of transcription (Chromosome 11 (green)) and distributes throughout the nucleus. (G) Tig1 cells: *NEAT1 *RNA (green) and *NEAT2/MALAT-1 *RNA are associated in the interphase nucleus. (H) MEFs: Mouse *Neat1 *RNA (red) is exclusively nuclear, and is found in fewer, larger foci than in human cells, yet shows the same peripheral association with *Neat2/Malat-1 *RNA (green), and neither RNA associates significantly with DAPI dense heterochromatin (blue).

In mouse and human cells, *NEAT1/Neat1 *displays a highly unusual localization pattern; *NEAT1*/*Neat1 *localizes to multiple large, bright foci which are often widely distributed (Figure 5A, E, G and 5H). In some cells, these foci have a more restricted distribution, showing polarity in two regions of many cells (Figure 5B). These *NEAT1 *RNA foci show sharply defined borders and little nucleoplasmic signal. RNA FISH studies show no evidence that the >17 kb *NEAT1 *transcript localizes in separate foci from the smaller ~4 kb transcript (data not shown). While the size and number of *NEAT1 *foci vary within an individual cell population, human cells tend to have more (~30 on average) *NEAT1 *foci that are smaller (~0.25 μm); whereas mouse cells have fewer (~7 on average), larger (~0.5 μM) *Neat1 *foci (see Figure 5G and 5H for comparison).

*NEAT2/MALAT-1 *shows a broad distribution throughout the nucleus. In many cells *NEAT2/MALAT-1 *is concentrated in ten to twenty distinct "domains" (Figure 5C, F, G and 5H), although weaker nucleoplasmic signal is also detected. The mouse cell lines examined show a mixed distribution: many cells have *Neat2/Malat-1 *RNA concentrated in domains, while other cells in the same population have only strong nucleoplasmic signal (Figure 5D).

**Figure 6:**
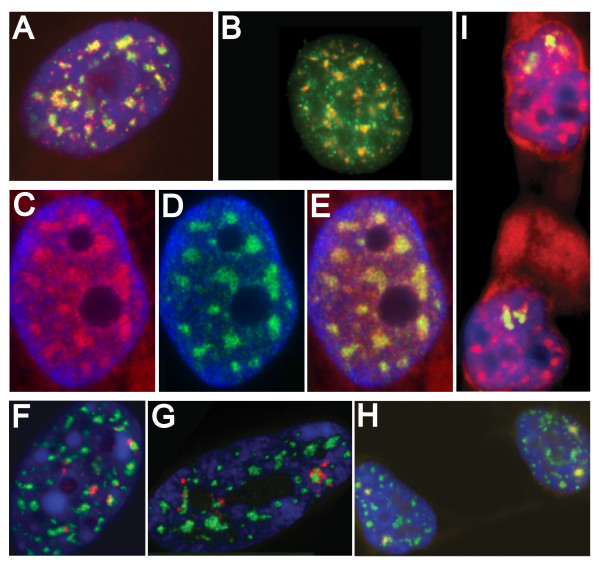
**Human and mouse *NEAT1 *and *NEAT2/MALAT-1 *are intimately associated with SC35 domains**. (A) Tig1 cells: *NEAT2*/MALAT-1 RNA (red) is found in every SC35 domain (green); (B) NIH-3T3s: *Neat2/Malat-1 *RNA (green) concentrates in domains in about 50% of the population, when it does, it overlaps with SRM-300 speckles (red); (C,D,E) *NEAT2/MALAT-1 *RNA (green (C)) and a probe to poly(A) (red (D)) overlap (yellow (E)) in domains in the nucleus; (F) *Neat1 *RNA (red) preferentially localizes to the periphery of SRM-300 domains (green) in mouse cells; (G) *NEAT1 *RNA (red) preferentially associates at the periphery of SC35 domains (green) in human cells; (H) Two transcription sites of *NEAT2/MALAT-1 *RNA (red) localize with SC35 domains (green) in early G1 daughter cells; (I) Poly(A) domains (red) are apparently formed prior6=- to the spread of *NEAT2/MALAT-1 *RNA (green) in early G1.

Early G1 daughter cells show two sites of transcription for both *NEAT1 *and *NEAT2/MALAT-1 *(Figure 5B and Figure 6H and 6I) and simultaneous gene/RNA localization (not shown) indicates they are both biallelically expressed. In contrast to *XIST*, *NEAT2/MALAT-1 *RNA shows no preferential association with its parental chromosome [5] (Figure 5F). Although *NEAT1 *RNA foci show a greater localization near chromosome 11 than does *NEAT2/MALAT-1 *RNA, *NEAT1 *transcripts do move away from their parent chromosome and distribute more broadly in the nucleus (Figure 5E).

Examination of the relationship between *NEAT1 *RNA foci and *NEAT2/MALAT-1 *RNA foci reveals that they do not overlap significantly in any cell type, but have a non-random and close association. In both human and mouse cells, the majority of *NEAT1 *foci preferentially localize to the periphery of *NEAT2/MALAT-1 *domains; oftentimes multiple *NEAT1 *foci encircle the *NEAT2/MALAT-1 *domain (Figure 5G and 5H).

### The Relationship Between *NEAT1 *and *NEAT 2 *RNAs and SC35 Domains

As *NEAT1 *and *NEAT2/MALAT-1 *transcripts are distributed across the nucleus in discrete domains, we asked whether these domains overlap with previously described nuclear structures. Our initial analyses indicate that the domain distribution of *NEAT2/MALAT-1 *RNA is similar to nuclear structures called SC35 splicing domains. As previously discussed, these structures are enriched in poly(A) RNA and factors involved in processing, splicing and export of mRNA. Many snRNAs concentrate within these domains and several individual mRNAs have been shown to enter the domains with which their genes associate upon transcription. However, while hypothesized for many years, no specific large poly(A) species, coding or noncoding, has been shown to be a component of all domains. We asked whether *NEAT2/MALAT-1 *was a component of these domains by performing RNA FISH in combination with immunohistochemical labeling with an antibody to SC35 in human cells and SRM-300 in mouse cells. Strikingly, in interphase cells with the typical domain pattern, *NEAT2*/*Neat2 *RNA is found in every SC35/SRM-300 domain (Figure 6A and 6B). While *NEAT2/MALAT-1 *RNA is clearly a component of these domains, there is not precise overlap: *NEAT2/MALAT-1 *RNA tends to concentrate more in the center of the domain but also defines a larger domain than either SC35 or SRM-300 (Figure 6B). SC35 was previously shown to form a smaller inner core within a larger domain defined by poly(A) RNA and Sm-defined speckles [37].

We next colocalized *NEAT1/Neat1 *RNA with SC35/SRM-300 in mouse and human cells (Figure 6F and 6G). Unsurprisingly, given their localization relative to *NEAT2/MALAT-1 *foci, *NEAT1 *foci predominantly border the domains, with multiple foci oftentimes encircling a single domain, especially in human cells.

As it has been speculated that a structural poly(A) RNA may be at the core of SC35 domains we first compared the distribution of poly(A) RNA and *NEAT2/MALAT-1 *RNA in mouse and human cells. These analyses revealed that poly(A) RNA and *NEAT2/MALAT-1 *transcripts were completely coincident in nuclear domains (Figure 6C, D and 6E). Immediately after mitosis there is a short window of time in which the SC35 (Figure 6H) and poly(A) (Figure 6I) domains are clearly reformed, but *NEAT2/MALAT-1 *RNA only overlaps the domains close to its transcription site. Shortly after in G1, *NEAT2/MALAT-1 *quickly distributes to all domains. These results show that while *NEAT2/MALAT-1 *RNA is a component of SC35 domains in most cells, it is not necessary for their formation.

## Discussion

In a microarray screen for nuclear, polyadenylated noncoding RNAs, we identified three abundant transcripts with significant nuclear retention: the canonical ncRNA, *XIST*, and two large noncoding loci on human chromosome 11. We show that these two ncRNAs are differentially expressed in a wide range of tissues in human and mouse and localize to specific subnuclear domains that have been the subject of much interest in relation to mRNA metabolism.

Our rationale for examining the nucleus for enriched poly(A) transcripts was prompted by two areas of research. First, the chromosome-wide regulation of monoallelically expressed genes (a potential autosomal analog of X inactivation) [38,39] suggests that there may be regulatory RNAs similar to *XIST *that interact with specific autosomes. Second, earlier biochemical studies indicated that there is a substantial population of heterogeneous nuclear RNA that does not give rise to cytoplasmic RNA [14] and it has long been suggested that this nuclear-retained RNA may provide insights into nuclear structure [11,40,41].

Recent genomic studies have identified a large number of putative ncRNAs based on analysis of ESTs (reviewed in [1]), however these approaches provide little insight into the biology of the many RNAs identified. Here we used a unique approach to identify poly(A) ncRNAs that met specific criteria of abundance and, most importantly, nuclear enrichment. Using Affymetrix arrays to compare the expression levels of over 39,000 transcripts in nuclear and cytoplasmic RNA fractions from two human cell-types, we identified transcripts from two loci, which like *XIST *are significantly enriched in the nucleus. These two loci are located less than 70 kb apart, yet represent two different genes with no homology to each other. *NEAT1 *and *NEAT2/MALAT-1 *are conserved within the mammalian lineage, yet neither contains conserved open reading frames. While *NEAT1 *contains two segments of high conservation, the ~8 kb sequence of *NEAT2/MALAT-1 *is highly conserved without insertions or deletions. This high level of conservation is particularly striking in light of the relative lack of conservation typically seen for large ncRNAs [32]. In fact, *NEAT2/MALAT-1 *appears to be more conserved than the canonical noncoding RNA *XIST*. While there are reports of both a larger, less abundant transcript and of a much smaller trophoblast specific transcript (TncRNA) that map to the human *NEAT1 *locus [28,29,31], both of these transcripts lie outside any region of conservation. Both human and mouse homologs of *NEAT1 *and *NEAT2/MALAT-1 *show broad, differential expression patterns across many different tissues. Further, in both organisms, of the over 39,000 transcripts examined in human and the over 79,000 transcripts examined in mice, *NEAT1*/*Neat1 *and *NEAT2*/*Neat2 *are among the most nuclear enriched poly(A) transcripts in multiple cell lines. Taken together, these findings of high conservation, broad expression and nuclear enrichment strongly suggest that *NEAT1 *and *NEAT2/MALAT-1 *have important functional roles within the mammalian nucleus.

The subnuclear distribution of *NEAT2/MALAT-1 *RNA provides immediate insights into its function. The broad distribution of *NEAT2/MALAT-1 *RNA clearly indicates that it is not involved in chromatin regulation of its parent chromosome. SC35 splicing domains (or speckles) are essentially universal components of the nuclei in higher eukaryotes. A number of specific protein coding genes have been identified which preferentially localize to the periphery of an SC35 domain in each cell, and for several of these their transcripts localize within those particular domains (reviewed in [10]). These domains concentrate numerous splicing factors, SR proteins, poly(A) RNA and mRNA export factors [8,10,42]. The concentration of *NEAT2/MALAT-1 *RNA in these domains thus strongly suggests it has a function related to pre-mRNA metabolism.

As previously mentioned, a large pool of polyadenylated (poly(A)) RNA localizes to each domain [13,15] and the identity of this poly(A) component has implications for the function of these structures. While it has long been speculated that some [11,16] or possibly all [13] of the poly(A) RNA in these regions may comprise a long-lived structural RNA, the poly(A) component of these domains might also be a heterogeneous mixture of pre-mRNA or mature RNA transcripts at their sites of maturation. While this latter condition would indicate that SC35 domains function as active sites of RNA processing and export, if this poly(A) component is composed of a small number of structural noncoding RNAs, this might instead suggest that SC35 domains function as storage sites for splicing factors. Importantly, such stable localization of ncRNAs to these domains could not only indicate a role for ncRNA in establishing the structure of SC35 domains but might also indicate that these ncRNAs contribute to the function of pre-existing domains either through their own enzymatic activity or by targeting specific proteins or RNAs to those domains.

While SC35 domains contain high concentrations of snRNAs, no large poly(A) RNA has been previously identified as a component of these domains. The presence of *NEAT2/MALAT-1 *in these regions is thus of substantial interest. Since *NEAT2/MALAT-1 *exhibits a pan-localization to every SC35 domain in most cells, we surmised that it may serve as a structural scaffold involved in the initial formation of these domains. However, our observations in early post-mitotic daughter cells argue against such an interpretation: domains in these cells clearly contain SC35 and poly(A) RNA, whereas *NEAT2/MALAT-1 *RNA is localized to just two sites of transcription. Thus, while *NEAT2/MALAT-1 *RNA may be a component of the poly(A) RNA in these domains, it does not account for all of it, consistent with evidence of other poly(A) pre-mRNAs in these regions. This does not rule out a later structural role for *NEAT2/MALAT-1*, but suggests it is more likely important for the function of pre-existing domains, rather than establishing the initial structure of these domains.

The identity of this remaining poly(A) signal present at the formation of SC35 domains is not known but is suggested by our studies. Insight into the nature of this RNA can be derived from the fact that no other poly(A) transcripts were identified as nuclear enriched in our analyses of the human and mouse transcriptomes. Given the nature of our study, we would have expected to find any highly expressed, nuclear retained, long ncRNAs which act in the formation of SC35 domains. Thus, our results suggest that the remaining poly(A) population present at the formation of SC35 domains is either short, poorly expressed, heterogeneous in nature, or not nuclear retained.

Our results indicate that there are at least two distinct populations of poly(A) RNA within SC35 domains. *NEAT2/MALAT-1 *is highly expressed and present in all mature SC35 domains, whereas the examination of nascent SC35 domains indicates the existence of a second population of other poly(A) RNAs. Further experiments will be necessary to determine the exact extent to which *NEAT2/MALAT-1 *RNA contributes to the overall poly(A) RNA signal in SC35 domains. However, if *NEAT2/MALAT-1 *comprises the bulk of the poly(A) component of SC35 domains its properties might obscure the properties of the remaining poly(A) signal. Thus, while many studies have made general observations concerning the properties of the bulk poly(A) component of SC35 domains, our results caution against over interpretation of these results. Directed study of *NEAT2/MALAT-1 *should help place these earlier observations in a clearer context.

The finding that *NEAT1 *RNA clusters typically localize at the edges of SC35 domains, regions known to be enriched in active genes [43,44], suggests a possible relationship of *NEAT1 *to pre-mRNA metabolism, however, in this case the implication is less clear. The observation that *NEAT1 *foci are often limited to certain nuclear regions does not appear to reflect a confinement to its parent chromosome, but likely reflects an affinity for other nuclear structures of limited mobility. Studies examining the relationship of *NEAT1 *RNA to other nuclear structures are ongoing (Clemson et al., in preparation), and preliminarily indicate that *NEAT1 *foci may have a complex relationship to a subset of paraspeckles, nuclear compartments which localize to the edge of speckles and contain components involved in adenosine to inosine substitution [45].

The overexpression of a transcript at the human *NEAT2 *locus, *MALAT-1*, has been associated with metastatic lung adenocarcinoma [30] but whether this is causative or merely correlative has not been established. Supporting our results, during the preparation of this manuscript the overexpression of a transcript corresponding to murine *Neat1 *termed *Vinc *(Virus-inducible noncoding RNA) was described in rabies infection and shown to be nuclear enriched in mouse renal adenocarcinoma cells (Figure 2B) [46]. To reflect its enrichment in the nucleus of many different cell types in different organisms and its homology to human NEAT1, we refer to the *Neat1*/*Vinc *transcript as *Neat1*. Given their broad expression and localization in ubiquitous nuclear structures, any dissection of the function of *NEAT1*/*Vinc *and *NEAT2*/*MALAT-1 *will require the examination of these RNAs in the context of a variety of cell types and conditions.

Aside from its utility in identifying two unique nuclear localized noncoding RNAs, the data from our screens yields other intriguing results when examined as a whole. For instance, our screens reveal the nuclear enrichment of RNA species that code for proteins with known nuclear localization patterns. Such enrichment may be indicative of nuclear translation, or translation of these proteins in the perinuclear space. Additionally, these studies identified several intronic probes in the Affymetrix dataset (see Additional file 1). As these probes are all transcribed from the same strand as the gene within which they reside and there is no reported bias for intronic genes to be transcribed in the same direction as the overlapping transcript, it appears that these probes detect intronic RNA rather than novel genes. This grouping of intronic probes might be of use in some interesting large scale splicing analyses. For instance, given that many of these intronic probes have corresponding exonic probes also represented in the U133A and B chipsets, analysis of existing Gene Expression Omnibus datasets for changes in the relative representation of these probes across samples might uncover conditions, cell types, or genetic backgrounds which display differential patterns of RNA splicing with respect to these genes.

## Conclusion

Our genome-wide screens in two mammalian species suggest that there are no more than three abundant large non-coding polyadenylated RNAs in the nucleus; the canonical large noncoding RNA *XIST, NEAT1 *and *NEAT2/MALAT-1*. A function for *NEAT1 *and *NEAT2/MALAT-1 *in mRNA metabolism is suggested by their high levels of conservation and their intimate association with SC35 splicing domains in multiple mammalian species. We have taken advantage of the power of genome-scale expression analysis and publicly available transcriptome data to study the subcellular localization of RNA species. By these broad approaches we have precisely identified noncoding RNA components of an enigmatic nuclear structure. The identification of specific poly(A) RNA species within SC35 domains should greatly facilitate the functional dissection of these domains. Further cytological analyses of the relationship of *NEAT1 *and *NEAT2/MALAT-1 *to pre-mRNA metabolism and specific nuclear structures is ongoing. Inhibition of these RNAs, through knockout or RNA-mediated interference (RNAi), may yield further clues as to their role in nuclear architecture or cell function and may ultimately facilitate the functional dissection of the nuclear structures with which they associate.

## Methods

### Subcellular fractionation of RNA and array analysis

WI-38 primary human fibroblasts (ATCC) and human EBV-transformed human lymphoblasts, GM00131 (Coriell) were grown under normal conditions. Nuclei were purified in triplicate from each cell line, using the Nuclei PURE kit (Sigma Aldrich). RNA from these nuclei was purified using RNeasy Maxi kit (Qiagen), along with RNA from cytoplasmic fractions. RNA representing equivalent numbers of cell equivalents was run on HGU133A and HGU133B Affymetrix expression arrays. For details concerning access to CEL file information see Additional file 4. Microarray data was normalized using Gene Cluster 2.0 [18] and filtered using a lower nuclear expression level threshold of 100. Probe sets showing more than 2-fold average higher nuclear expression levels as compared to cytoplasm expression levels in both cell types were aligned to the human genome and qualified into separate transcript type categories using the University of California at Santa Cruz genome browser.

### RACE and Splicing analysis

5'/3'-RACE was performed on RNA from WI-38's, GM00131's, mouse NIH 3T3 fibroblasts, and mouse primary embryonic fibroblasts using the FirstChoice RLM-RACE Kit (Ambion). Gel purified products were TOPO cloned (Invitrogen) and sequenced. For details of splicing analysis see Additional file 4.

### Mouse transcriptome analysis

Datasets analyzed were taken from the NCBI Gene Expression Omnibus with accession numbers: BLK CL.4 cells: nuclear RNA – GSM17241, cytoplasmic RNA – GSM17242; Liver: nuclear RNA – GSM17244, cytoplasmic RNA – GSM17245. Tag counts were normalized to the total number of tags counted per experiment and averaged for each experimental condition. Tags were filtered for more than 10 fold higher average tag count in nuclear versus cytoplasmic samples from both cell types and mapped to transcripts using BLAT [19].

### Northern blot analysis

Commercially obtained Northern blots (Ambion's FirstChoice Human Blot 2 and Mouse Blot 1) were examined for the presence of human and mouse *NEAT1 *and *NEAT2/MALAT-1 *according to the manufacturer's instructions. Probes for Northern analysis were amplified from genomic DNA for *NEAT1 *from human chr11:64946880-64947322 (May 2004 build), with primers hNEAT1NP1F (5'-TAGTTGTGGGGGAGGAAGTG-3') and hNEAT1NP1R (5'-TGGCATGGACAAGTTGAAGA-3'), for human *NEAT2/MALAT-1 *from human chr11:65,029,292-65,029,727 (May 2004 build) with primers hNeat2NP2F (5'-GGCAGGAGAGACAACAAAGC-3') and hNeat2NP2R (5'-CCTCGACACCATCGTTACCT-3'), for mouse *Neat1 *from mouse chr19:5,739,363-5,739,846 (Aug 2005 build) with primers mNeat1NP1F (5'-CAGGGTGTCCTCCACCTTTA-3') and mNeat1NP1R (5'-AAACCAGCAGACCCCTTTTT-3') and for mouse *Neat2/Malat-1 *from mouse chr19:5,691,020-5,691,420 (Aug 2005 build) with primers mNeat2NP1F (5'-GTTACCAGCCCAAACCTCAA-3') and mNeat2NP1R (5'-CTACATTCCCACCCAGCACT-3'). The human *NEAT1 *Northern probe PCR product was TOPO cloned into pCR4TOPO (Invitrogen) and the construct linearized with NotI. Probe for human *NEAT1 *was labeled with (α-32P)UTP by T3 RNA polymerase with the Ambion Strip-EZ RNA probe synthesis kit according the manufacturer's instructions. Human *NEAT2/MALAT-1*, mouse *Neat1 *and mouse *Neat2/Malat-1 *Northern probe PCR products were gel purified and labeled with (α-32P)dCTP by Klenow with the Ambion Strip-EZ DNA probe synthesis kit according to manufacturer's instructions. Labeled probe was hybridized to the blot using the Ambion NorthernMax formaldehyde-based system for Northern blots according to the manufacturer's instructions.

### Comparison of NEAT1 and NEAT2/MALAT-1 between the human and mouse genomes

EMBOSS Dotmatcher was used to compare the genomic regions spanned by *NEAT2/MALAT-1, NEAT1*, and *XIST *in humans with their homologs on mouse chromosome 19 and X as well as to compare human *NEAT1 *with human *NEAT2/MALAT-1*. A sliding window size of 25 nt and an identity cutoff of 80% were used for interspecies comparisons to highlight regions of increased conservation while an identity cutoff of 50% was used for the intraspecies comparison to highlight the lack of conservation.

### Cells and Cell Preparation Techniques for In Situ Hybridization

Normal human female diploid fibroblast cells TIG1 (Coriell); mouse fibroblasts NIH-3T3 (ATCC); primary mouse embryo fibroblasts (MEFs) (Cell Essentials) were cultured under normal conditions.

Cells were prepared for FISH as previously described [20,21] along with two modified techniques to rigorously preserve cytoplasmic RNA. In one, the triton extraction was reduced to 30 seconds prior to fixation; in the other, cells were fixed in 4% paraformaldehyde prior to a 5 minute triton extraction. Cells were prepared for whole chromosome library hybridization as described previously [7].

### FISH and Protein Immunofluorescence

Probes used: *XIST*: G1A: genomic plasmid that spans 10 kb from intron 4; Chromosome 11: Cambio Starfish Human Whole Probe (Open Biosystems). *NEAT1/Neat1 *and *NEAT2/Neat2 *probes (>3 kb) were generated by PCR against the following genomic regions: ~4 kb human *NEAT1 *transcript – human chr11:64946934-64950629 (Mar 2006 build);) with primers hNEAT1_shortprobe_F (5'-CAAGTCCAGCCGGAGTTAGCGACAG-3') and hNEAT1_shortprobe_R (5'-ATGAAGGCAATGTGATAGGGGTCGAGA-3'); the >17 kb kb human *NEAT1 *transcript- human chr11:64950063-64954198 (Mar 2006 build) with primers 17hNEAT1_probe1_F (5'-CTAAAAAGGGAAGGGGATGGGGATTGT-3') and 17NEAT1_probe1_R (5'-CATTTACCCGCATTTCACAGACACAGG-3');and human *NEAT2/MALAT-1 *– human chr11:65023715-65027427 (Mar 2006 build) with primers hNEAT2_probe_F (5'-GGAAGACAGAAGTACGGGAAGGCGAAG-3') and hNEAT2_probe_R (5'-CATCACTGAAGCCCACAGGAACAAGTC-3'). The mouse *Neat2/Malat-1 *transcript probes were generated by PCR with Advantage 2 polymerase (BDClontech) against mouse chr19:5796993-5800515 (Aug 2005 build) from genomic DNA with primers mNEAT2_region_F (5'-CAGGATAAGCAGAGCTCGCCAGGTTTA-3') and mNEAT2_region_R (5'-GGCTCGTTCACCTGTTGTCCTCATTTT-3') and TOPO cloned into pCRII-TOPO (Invitrogen). The mouse *Neat1 *transcript probe was generated by PCR against mouse chr19:5842302-5845478 (Aug 2005 build) from cDNA with primers 3'endmNeat1 (5'-GAAGCTTCAATCTCAAACCTTTA-3') and 5'endmNeat1 (5'-AGGAGTTAGTGACAAGGAG-3') and TOPO cloned into pCR4TOPO (Invitrogen).

The direct PCR products or plasmids containing the PCR products or genomic clones were purified and nick translated with digoxigenin-11-dUTP or biotin-16-dUTP (Roche). Our protocols for FISH and immunofluorescence, as well as combined in situ RNA and antibody detection, have been described previously in detail [10,20,21], with antibody detection normally carried out prior to hybridization. SC35 domains were detected with an antibody to the spliceosome assembly factor SC35 [9] (Sigma) or to an antibody to the splicing coactivator SRM300 [22] (B. Blencowe, U. Toronto).

Poly(A) RNA was detected using a biotin-labeled poly dT(55mer) oligonucleotide. Oligo hybridization was performed for 3 hours at 37°C. The initial wash was performed at reduced stringency: 10% formamide/2X SSC for 10 minutes at 37°C. RNA detection with whole chromosome hybridization was performed as recently described [7].

### Oligonucleotides

Oligonucleotide primers were designed using Primer3 and ordered from Integrated DNA Technologies (IDT). For any sequences not specifically mentioned here, see Additional file 4.

## Abbreviations

ncRNA – noncoding RNA

FISH – fluorescent in situ hybridization

NEAT – nuclear enriched abundant transcript

## Authors' contributions

JH and AWE carried out the molecular and genomic analyses. CMC performed the cytological analyses with help from CRL. AC and JBL were involved in the design of experiments, along with the other authors. All authors participated in writing the manuscript. All authors read and approved the final manuscript.

## Supplementary Material

Additional File 1 Categories of Affymetrix probes over 2-fold enriched and with a cytoplasmic intensity cutoff above 100 in both GM00131 lymphoblast and WI-38 fibroblast lines. Click here for file

Additional File 2 Figure describing splicing analysis of *NEAT1 *and *NEAT2*. Click here for file

Additional File 3 MPSS tags and gene descriptions over 2-fold enriched in both mouse liver and BLK CL.4 cell lines. Click here for file

Additional File 4 Detailed information regarding array analysis of nuclear and cytoplasmic RNA. Detailed information regarding splicing analysis of *NEAT1 *and *NEAT2*. Click here for file

## Figures and Tables

**Table 1:** Murine transcripts showing more than 10 fold nuclear enrichment in both BLK CL.4 and Liver cells

	**Fold Nuclear Enrichment**	
**Transcript**^**a**^	**BLK CL.4**	**Liver**
*Neat2*	80.8	17.4
*Neat1*	42.8	19.5
*Pabpn1*	38.5	48.9
*Fnbp4*	19.0	38.4
*Cd47*	17.2	33.9
*Plec1*	11.6	16.4

^a ^Transcripts are ranked in descending order of nuclear enrichment in BLK CL.4 cells from top to bottom.
