# Supplementary material for: A screen for nuclear transcripts identifies two linked noncoding RNAs associated with SC35 splicing domains
Source: BMC Genomics. 2007 Feb 1;8:39. doi: 10.1186/1471-2164-8-39 (PMC1800850; doi:10.1186/1471-2164-8-39)
Supplement: Additional File 2 — Figure describing splicing analysis of NEAT1 and NEAT2. [file 1471-2164-8-39-S2.pdf]

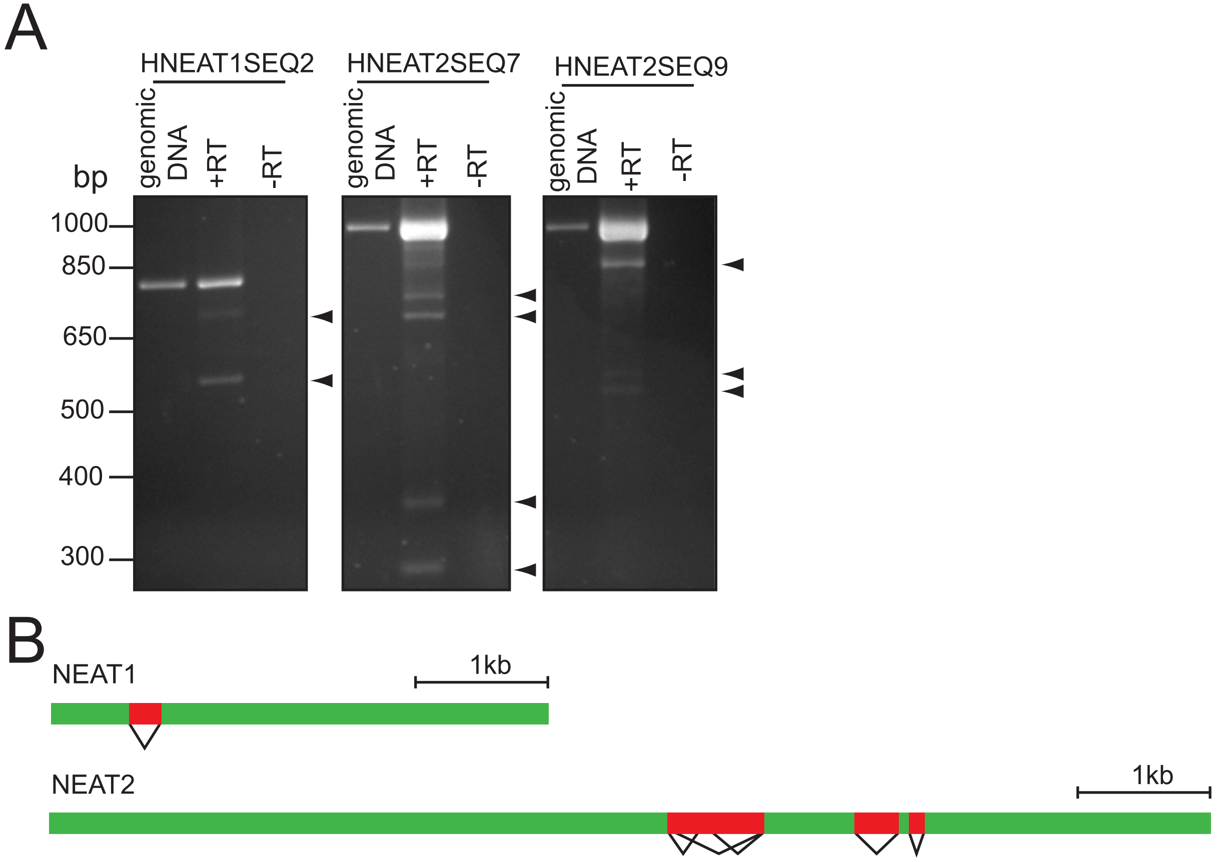

Supplementary Figure S1 – Splicing of the Human NEAT1 and NEAT2 transcripts. **(A)** To look for introns within the NEAT1 and NEAT2 transcripts, RNA from GM00131 EBV transformed lymphoblasts (Coreill) was reverse transcribed with random primers and PCR amplified at overlapping ~750-1000 nucleotide intervals along the predicted transcripts. All cDNA amplification samples were compared to matched genomic amplifications by agarose gel electrophoresis. While most primer sets resulted in the amplification of identical bands from genomic DNA or cDNA, one primer set for NEAT1 (HNEAT1SEQ2) and two for NEAT2 (HNEAT2SEQ7 and HNEAT2SEQ9) amplified smaller products in the RT-PCR samples than in the genomic PCR samples. These potential intron containing regions were amplified (as indicated by arrows) at a lower frequency than the full size, unspliced product indicating that the majority of these transcripts are unspliced. Negative control samples without Superscript III in the reaction (-RT) confirm lack of genomic DNA contamination within the cDNA samples. **(B)** Introns with consensus splice acceptor sites GT-AG or GC-AG were mapped to the transcript, shown here graphically in red with lines indicating intron limits.
