# Supplementary material for: A screen for nuclear transcripts identifies two linked noncoding RNAs associated with SC35 splicing domains
Source: BMC Genomics. 2007 Feb 1;8:39. doi: 10.1186/1471-2164-8-39 (PMC1800850; doi:10.1186/1471-2164-8-39)
Supplement: Additional File 4 — Detailed information regarding array analysis of nuclear and cytoplasmic RNA. Detailed information regarding splicing analysis of NEAT1 and NEAT2. [file 1471-2164-8-39-S4.pdf]

Supplementary Methods:

Detailed information regarding array analysis of nuclear and cytoplasmic RNA:

Scans as raw CEL files are available at http://www.broad.mit.edu/cgi-bin/cancer/web_tools/get_files/select_cel.cgi and are named as follows:

Fibroblasts (WI-38) - Cytoplasmic RNA:

HGU133A - CL2003010801AA, CL2003011002AA and CL2003010803AA.

HGU133B - CL2003011401AA, CL2003011402AA and CL2003011403AA.

Fibroblasts (WI-38) - Nuclear RNA:

HGU133A - CL2003010804AA, CL2003010805AA and CL2003010806AA.

HGU133B - CL2003011404AA, CL200301405AA and CL2003011406AA.

Lymphoblasts (GM00131) - Cytoplasmic RNA:

HGU133A - CL2003020701AA, CL2003020702AA and CL2003020703AA.

HGU133B - CL2003020707AA, CL2003020708AA and CL2003020709AA.

Lymphoblasts (GM00131) - Nuclear RNA:

HGU133A - CL2003020704AA, CL2003020705AA and CL2003020706AA.

HGU133B - CL2003020710AA, CL2003020711AA and CL2003020712AA.

Detailed information regarding splicing analysis of NEAT1 and NEAT2

To look for introns within the *NEAT1* and *NEAT2* transcripts, RNA from GM00131 cells was reverse transcribed with random primers and PCR amplified at overlapping ~750-1000 nucleotide intervals along the predicted transcripts. All cDNA amplification samples were compared to matched genomic amplifications by agarose gel electrophoresis and matched products sequenced. Smaller products were gel purified, TOPO cloned and sequenced. Sequences with splice acceptor sites of GT-AG or GC-AG were mapped to the transcript.

cDNAs were amplified at overlapping ~750-1000 nucleotide intervals with the following primer pairs:

For *NEAT1:*

hNeat1seq1F (5’-GTTAGCGACAGGGAGGGATG-3’) and hNeat1seq1R (5’-GCGCCTTAACTCCACATCA-3’)

hNEAT1SEQ2F (5’-AACTTGTCCATGCCAGCAG-3’) and hNEAT1SEQ2R (5’-CTCTCTCCTCCAGGGTCTCC-3’)

hNEAT1SEQ3F (5’-GTGGCTGTTGGAGTCGGTAT-3’) and hNEAT1SEQ3R (5’-TCAACGCCCCAAGTTATTTC-3’)

hNEAT1SEQ4F (5’-TTGTTCCAGAGCCCATGAAT-3’) and hNEAT1SEQ4R (5’-TTCCCTTCAACCTGCATTTC-3’)

hNEAT1SEQ5F (5’-AGAAGGGAATGGTGGGTACA-3’) and hNEAT1SEQ5R (5’-TCAAATCAACCACCTAAGTTGC-3’)

hNEAT1SEQ6F (5’-CAAGGTGGGGAAGACTGAAG-3’) and hNEAT1SEQ6R (5’-CCATCCCCTTCCCTTTTTAG-3’)

hNEAT1SEQ7F (5’-AAATCGTGCCTTAGAAACACATC-3’) and hNEAT1SEQ7R (5’-GTTTAGAACTCAAACTTTATTTGTGC-3’)

For *NEAT2:*

HNEAT2SEQ1F (5’-GGCCTCTCCTGCCCTCTTA-3’) and HNEAT2SEQ1R (5’-AACGGGTCATCAAACACCTC-3’) and HNEAT2SEQ2F (5’-AGATTTCCCAAGCAGACAGC-3’)

HNEAT2SEQ2R (5’-AAGCTGTTTAAGTCACCTTCATTTT-3’) and HNEAT2SEQ3F (5’-GCTTGAGGAAACCGCAGATA-3’)

HNEAT2SEQ3R (5’-AAACACCCTCATCTTCTCAAGC-3’) and HNEAT2SEQ4F (5’-AATTGGATAAAATAGCACTGAAAAA-3’)

HNEAT2SEQ4R (5’-GCCCTCAAAAGCTTCAGACA-3’) and HNEAT2SEQ5F (5’-ACAGGGAAAGCGAGTGGTT-3’)

HNEAT2SEQ5R (5’-CAAAGCTGCACTGTGCTGTA-3’) and HNEAT2SEQ6F (5’-AAAGTGCTTAACCCCTTAAACTTG-3’)

HNEAT2SEQ6R (5’-TGTTTTGCAGTTAAACAATGGAA-3’) and HNEAT2SEQ7F (5’-GGCCTACTGGGCTGACATTA-3’)

HNEAT2SEQ7R (5’-CTGCCTGCTGTTTTCTGCT-3’) and HNEAT2SEQ8F (5’-CAAAAGCAGAATAAAAGCGAAAA-3’)

HNEAT2SEQ8R (5’-CCCCTGACTTTCTGGAAATAAA-3’) and HNEAT2SEQ9F (5’-TCTGCGAACACTCTTTAATGGA-3’)

HNEAT2SEQ9R (5’-TGTTGCTTGTTTGGAATGTTTC-3’) and HNEAT2SEQ10F (5’-TCCCCAATGCTTGGAGTAGT-3’)

HNEAT2SEQ10R (5’-CGCTTGAGATTTGGGCTTTA-3’) and HNEAT2SEQ11F (5’-CTGTGGCAGGAGAGACAACA-3’)

HNEAT2SEQ11R (5’-GCACCTGCAGAGAAAAGGAG-3’) and HNEAT2SEQ12F (5’-GGCAGGAGAGACAACAAAGC-3’)

HNEAT2SEQ12R (5’-AGCACCTGCAGAGAAAAGGA-3’) and HNEAT2SEQ13F (5’-GAGGTCTTTGGTGGGTTGAA-3’) and HNEAT2SEQ13R (5’-GAGCTTCTCCATTTTTATTACACAA-3’)
